# Prevalence and Predominant Genotype of Hepatitis C Virus Infection and Associated Risk Factors among Pregnant Women in Iran

**DOI:** 10.1155/2021/9294276

**Published:** 2021-09-18

**Authors:** Fatemeh Farshadpour, Reza Taherkhani, Farkhondeh Bakhtiari

**Affiliations:** ^1^Department of Virology, School of Medicine, Bushehr University of Medical Sciences, Bushehr, Iran; ^2^Persian Gulf Biomedical Sciences Research Institute, Bushehr University of Medical Sciences, Bushehr, Iran

## Abstract

**Objective:**

Knowledge regarding the prevalence and risk factors of hepatitis C virus (HCV) infection among pregnant women can give clue to health care providers regarding the appropriate management of HCV infection. Therefore, this study was conducted to determine the prevalence, genotypic pattern, and risk factors of HCV infection among pregnant women in the northern shores of the Persian Gulf, south of Iran.

**Methods:**

From January 2018 to June 2019, serum samples were obtained from 1425 pregnant women, ages ranging from 14 to 46 years (28.1 ± 5.99). Serum samples were tested for detection of anti-HCV antibodies using an enzyme-linked immunosorbent assay (ELISA) (HCV Ab ELISA kit, Dia.Pro, Milan, Italy). Following the extraction of nucleic acid, the molecular evaluation of HCV infection was performed by seminested reverse transcriptase-polymerase chain reaction assay (RT-PCR), targeting the 5′ untranslated region (5′UTR) and core of HCV genome and sequencing.

**Results:**

Of the 1425 pregnant women, 19 women (1.33%, 95% CI: 0.85%–2.07%) were positive for anti-HCV antibodies. The majority of HCV-seropositive women were in the third trimester of pregnancy, educated, and had a history of blood transfusion, abortion, surgery, or dentistry. Moreover, Arab and Fars pregnant women and those aged >39 years had the highest rate of HCV seroprevalence. Nevertheless, none of these variables were significantly associated with HCV seropositivity. In contrast, HCV seropositivity was associated with place of residency, so that residents of Khormuj city had significantly higher HCV seroprevalence compared to the residents of other cities (OR: 7.05; 95% CI: 1.75–28.39; *P* = 0.006). According to the molecular evaluation, 9 of the 19 HCV-seropositive pregnant women (47.37%) had HCV viremia with genotype 3a.

**Conclusion:**

This study reports the HCV prevalence of 1.33% for anti-HCV antibodies and 0.63% for HCV RNA among pregnant women in the south of Iran. Considering the asymptomatic nature of chronic HCV infection and the fact that vertical transmission is possible in women with detectable viremia, therefore, screening of women before pregnancy is recommended to reduce the risk of HCV infection and its complications during pregnancy.

## 1. Introduction

Hepatitis C virus (HCV), a member of the family *Flaviviridae*, is one of the major causes of chronic liver disease. This positive-stranded RNA virus is predominantly transmitted through exposure to infected blood. The clinical manifestations of HCV infection vary from asymptomatic to acute self-limited or chronic infection, which might progress to cirrhosis and hepatocellular carcinoma within several years [1–4].

The global prevalence of HCV infection in children varies from 0.05% to 0.36% in developed countries and from 1.8% to 5% in developing countries [5]. After screening blood donors for hepatitis C, mother-to-child transmission has become the major cause of pediatric HCV infection [6]. There is a direct relationship between maternal viral load and vertical transmission of HCV. High maternal viral load increases the risk of infection in the offspring [5, 7]. If HCV RNA is present in the mother's blood, the rate of vertical transmission is about 4.3% [6, 8]. Moreover, in HIV-infected mothers, the rate of vertical transmission increases to 10.8% [9, 10]. Although the high maternal viral load is an important factor for the perinatal transmission of HCV, this risk factor is not preventable, as no anti-HCV treatment can currently be prescribed to pregnant women to prevent HCV from replicating [7]. Caution is essential in adopting delivery methods, amniocentesis, or internal fetal monitoring that can increase the fetus' exposure to HCV through infected maternal blood [5, 7].

Vertical transmission is almost always limited to women with detectable HCV viremia [11–13]. HCV prevalence in pregnant women is, to some extent, reflective of the prevalence in general population. Therefore, the rate of maternal-fetal transmission of HCV varies in different geographical regions. Universal HCV screening during pregnancy is recommended to provide the best pregnancy management interventions to reduce the risk of perinatal HCV transmission [10, 14]. The prevalence of HCV in Iran ranges from 0.5% (95% CI: 0.4–0.6%) in blood donors to 0.6% (95% CI: 0.4% to 0.8%) in the general population [15, 16]. However, despite the importance of HCV infection during pregnancy, the epidemiology of HCV infection among pregnant women remains unknown in most parts of Iran. Therefore, this study is aimed at determining the risk factors, prevalence, and genotypic pattern of hepatitis C among pregnant women residents in the northern shores of the Persian Gulf, south of Iran. This is the first report on the molecular identification of HCV infection among pregnant women in the south of Iran.

## 2. Material and Methods

### 2.1. Study Setting and Population

From January 2018 to June 2019, serum samples were obtained from 1425 pregnant women attending the public health centers for periodical checkups using the multistage cluster sampling method. In this method, five cities of the northern shores of the Persian Gulf, including Bushehr (with an overall population of 253024), Borazjan (230685), Jam (52990), Khormuj (80616), and Ahram (73637), and three of the most populated public health centers from each city located in Southern Iran were selected randomly. After describing the aim of the study, the pregnant women attending these public health centers were requested to participate in this study. During the study period, 1858 pregnant women referred to these public health centers; 1425 of whom agreed to participate in this survey. All pregnant women who agreed to participate and gave written informed consent to use their samples for HCV detection and their data for analysis were included consecutively in this study. The sociodemographic characteristics and pregnancy information were obtained from the participants during enrollment by interviewing using a questionnaire or the records of pregnant women at the public health centers. This study was funded by the Deputy Research and Affairs of Bushehr University of Medical Science with grant number 461 and was approved by the Ethical Committee of the University with reference number IR.BPUMS.REC.1396.117.

### 2.2. Laboratory Diagnosis

The serum samples were tested for detection of anti-HCV antibodies using an enzyme-linked immunosorbent assay (ELISA) (HCV Ab ELISA kit, Dia.Pro, Milan, Italy). The specificity and sensitivity of this kit were 99.5% and 100%, respectively. All seropositive samples were tested using seminested reverse transcriptase-polymerase chain reaction (RT-PCR) assay as described in our previous study [17]. Briefly, the nucleic acid was extracted from the samples using High Pure Viral Nucleic Acid kit (Roche, Mannheim, Germany) according to the manufacturer's instructions. Following the extraction of viral RNA, the molecular evaluation of HCV infection was performed by nested RT-PCR assay, targeting the 5′ untranslated region (5′UTR) and core of the HCV genome and sequencing. The extracted HCV RNA was reverse transcribed into cDNA using the SuperScript III cDNA synthesis kit (Invitrogen, Carlsbad, CA, USA). The cDNA was amplified by seminested PCR using outer primers (forward primer (–268 to –251): AGCGTCTAGCCATGGCGT; reverse primer (+410 to +391): ATGTACCCCATGAGGTCGGC) and inner primers (forward primer (–268 to –251): AGCGTCTAGCCATGGCGT; reverse primer (+383 to +364): CACGTTAGGGTATCGATGAC). The 680 bp and 580 bp length fragments of the HCV genome from the 5′UTR through core were amplified by these sets of seminested primers. The amplicons were sequenced to determine HCV genotypes.

### 2.3. Statistical Analysis

Data were analyzed by using SPSS 17 package program (SPSS Inc., Chicago, IL, USA) and were compared by Student's *t*-test and chi-square test or Fisher's exact test, and *p* values < 0.05 were considered statistically significant. Logistic regression analysis was used to determine the risk factors of HCV infection among pregnant women, and the odds ratio with 95% confidence intervals was calculated.

## 3. Results

Serum samples were obtained from 1425 pregnant women, including 616 participants from Bushehr city, 440 participants from Borazjan city, 207 participants from Ahram city, 122 participants from Jam city, and 40 participants from Khormuj city, with ages ranging from 14 to 46 years (28.1 ± 5.99). Of these, 108 (7.6%) participants were pregnant women less than 20 years old, 283 (19.9%) were 20–24 years old, 503 (35.3%) were 25–29 years old, 293 (20.6%) were 30–34 years old, 192 (13.5%) were 35–39 years old, and 46 (3.2%) were over 39 years old. Of 1425 pregnant women, 19 women (1.33%, 95% CI: 0.85%–2.07%) were positive for anti-HCV antibodies. The highest rate of anti-HCV seroprevalence was observed in the age group > 39 years (2.2%) followed by the age group 25–29 years (2.0%), whereas the lowest anti-HCV seropositivity was found in the age group < 20 years (0.9%) and the age group 20–24 years did not show anti-HCV seropositivity. Anti-HCV-seropositive pregnant women had a higher mean age (29.37 ± 5.64) compared to anti-HCV-seronegative pregnant women (28.09 ± 5.6), while this difference was not statistically significant (*P* = 0.35). Of the 19 anti-HCV-seropositive pregnant women, 17 women had normal levels (up to 32 U/L) of alanine transaminase (ALT) and aspartate transaminase (AST). Two anti-HCV-seropositive women had elevated levels of ALT and AST; one of these cases was viremic; this case was also positive for hepatitis B surface antigen (HBsAg) (ALT: 76 U/L and AST: 60 U/L). The other case was positive for anti-HCV antibodies but negative for HCV viremia (ALT: 45 U/L and AST: 38 U/L). Besides, all of the anti-HCV-seropositive samples were negative for HIV.

Regarding ethnicity and place of residency, Arab (1.5%) and Fars (1.4%) pregnant women and those residents of Khormuj city (7.5%) had the highest anti-HCV seropositivity, while residents of Ahram city (1.0%) and Afgan and Turk women showed the lowest rate of anti-HCV seroprevalence. The majority of anti-HCV-seropositive women were in the third trimester of pregnancy, educated, and had a history of blood transfusion, abortion, surgery, or dentistry. Nevertheless, anti-HCV seroprevalence among pregnant women was not statistically associated with the number of pregnancies, stage of gestation, age, ethnicity, level of education, time of sampling, history of abortion, blood transfusion, surgery, and dentistry. In contrast, anti-HCV seropositivity was associated with the place of residency, so that residents of Khormuj city had significantly higher anti-HCV seroprevalence compared to the residents of other cities (OR: 7.05; 95% CI: 1.75–28.39; *P* = 0.006).

**Figure 1:**
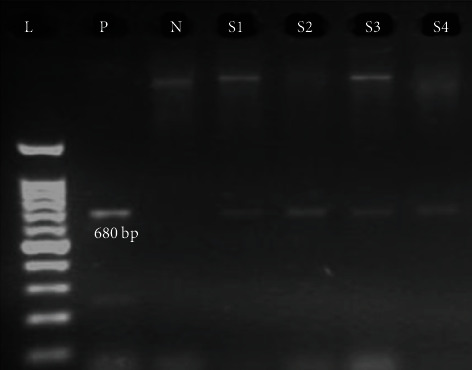
The first round of RT-PCR amplification of HCV RNA extracted from samples of pregnant women. L: 100 bp DNA ladder; P: positive control; N: negative control; S1–S4: amplified product (680 bp) on 2% agarose gel electrophoresis.

**Figure 2:**
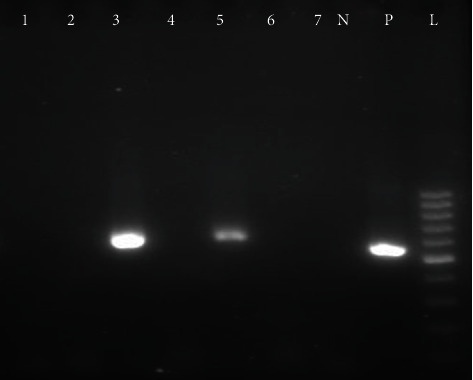
The second set of RT-PCR amplification of negative PCR products from first-round PCR. L: 100 bp DNA ladder; P: positive control; N: negative control; 3 and 5: amplified product (580 bp) on 2% agarose gel electrophoresis.

According to the molecular evaluation, 9 of 19 HCV-seropositive samples (47.37%) had HCV viremia with genotype 3a. So, 4 samples were found to be positive in the first round of PCR (Figure 1) and 5 samples were positive in the second round of PCR (Figure 2). Regarding sociodemographic characteristics and qualitative variables, no statistical difference was found between anti-HCV-positive samples with viremia and anti-HCV-positive samples without viremia. Overall, the prevalence of HCV viremia among pregnant women was 0.63% (95% CI: 0.33%–1.19%). The mean age of pregnant women with HCV viremia (31.0 ± 3.71) was higher than that of HCV RNA-negative pregnant women (28.09 ± 5.99), but this difference was statistically insignificant (*P* = 0.146). Except for the place of residency, almost no risk factor was found for HCV viremia among pregnant women. HCV viremia was significantly higher among pregnant women of Khormuj city (5%) than those of the other cities (1.65%) (OR: 10.75; 95% CI: 1.74–66.3; *P* = 0.010). The prevalence of anti-HCV antibodies and HCV viremia among pregnant women grouped according to sociodemographic characteristics and qualitative variables is presented in Tables 1 and 2.

## 4. Discussion

HCV infection, with a global prevalence rate of 2.8% and more than 350000 deaths annually, is considered to be the major causative agent of viral hepatitis-related mortality [1]. Nevertheless, the epidemiological pattern of HCV infection among pregnant women, one of the most vulnerable population groups, remains unknown in most parts of the world. Besides, no report is available on molecular identification and risk factors of HCV infection among pregnant women in the northern shores of the Persian Gulf, Iran. Therefore, in this survey, we investigated the prevalence and genotypic pattern of HCV infection among pregnant women in this territory and found the HCV prevalence of 1.33% for anti-HCV antibodies and 0.63% for HCV viremia with genotype 3a.

The HCV seroprevalence of 1.33% observed in the pregnant women is considerably higher than the HCV prevalence of 0.1% identified in the blood donors of this territory [18]. Besides, the HCV seroprevalence reported in this study is higher than those reported among the blood donors (0.5%) and the general population (0.6%) of Iran [15, 16]. Therefore, the pregnant population of this territory should be considered as the at-risk population. Moreover, all the HCV-infected pregnant women were unaware of their infection, which may be indicative of the asymptomatic nature of chronic HCV infection. Screening for HCV infection can result in an increase in the identification of infected but asymptomatic pregnant women and neonates born at risk. Therefore, detection of HCV infection should be included in the screening program of pregnant women to prevent those procedures that can increase the fetus' exposure to HCV and improve clinical management of infants born to infected mothers. Moreover, routine screening of women of childbearing age for HCV infection before pregnancy and antiviral treatment of HCV-infected patients can mitigate the risk of mother-to-child transmission and complications of HCV infection during pregnancy such as cholestasis and preterm birth [19, 20]. The prompt diagnosis and proper treatment of infected individuals as well as timely interventions and public education improve prevention and management strategies and, consequently, reduce the rate of infection in the community [1].

The seroprevalence of 1.33% for anti HCV antibodies reported in this study is higher than those reported among pregnant women in some parts of Iran, 0.98% in Malekan (northwest of Iran) [21] and 0.2% in Lorestan (west of Iran) [22]. The anti-HCV seroprevalence reported in the present study is also higher than those reported among pregnant women in Sweden (0.6%) [23], Central Sudan (0.6%) [24], Tanzania (0.3%) [25], Central Brazil (0.22%) [26], Saudi Arabia (0.07%) [27], Eastern Turkey (0.06%) [28], and Kurdistan Region of Iraq (0.04%) [29] but slightly lower than those of Ghana (7.7%) [30], Cameroon (1.9%) [31], Northeast Italy (1.9%) [32], Ethiopia (1.8%) [33], India (1.7%) [34], Egypt (1.7%) [35], Pakistan (1.42%) [36], and Nigeria (1.39%) [37]. These variations in the prevalence of HCV infection might be due to differences in HCV epidemiological patterns, risk factors, the routes of transmission, and general health status in different regions of the world. However, these variations can be explained by differences in sociodemographic characteristics of the study population, sample size, study period, time of sampling, and specificity and sensitivity of diagnostic methods in different studies.

In this study, the highest rate of anti-HCV seroprevalence was observed in the age group > 39 years followed by the age group 25–29 years. In our previous study among the blood donors of this territory, the highest rate of anti-HCV seroprevalence was reported in the age group 31 to 40 years followed by the age group 20 to 30 years [18]. Previous studies from the west and the northwest of Iran showed the highest rates of anti-HCV seropositivity among pregnant women aged < 20 years and 25 to 29 years, respectively [21, 22]. Besides, in this study, the mean age of HCV-seropositive pregnant women was higher than that of HCV seronegative pregnant women. This is in accordance with the age distribution of HCV in Southwest Ethiopia [38] and Egypt [39], where hepatitis C is more prevalent among older pregnant women. The high prevalence of HCV infection among older pregnant women is a cause for concern. Considering the complications of chronic HCV infection, preventive strategies, appropriate precautions, and training programs are recommended to reduce the incidence and adverse effects of HCV infection in the pregnant population.

According to the result of the present study, HCV seropositivity among the pregnant women was not statistically associated with the number of pregnancies, stage of gestation, age, ethnicity, level of education, time of sampling, history of abortion, blood transfusion, surgery, and dentistry, although the majority of HCV-seropositive women were in the third trimester of pregnancy, educated, and had a history of blood transfusion, abortion, surgery, or dentistry. Previous studies from the west and northwest of Iran revealed almost no risk factor for HCV seropositivity among pregnant women [21, 22]. The same findings have been reported in the study of Chibwe et al. [25]. In contrast, some studies from Egypt, Pakistan, and Ghana have reported a significant association between the history of blood transfusion and HCV seropositivity [30, 35, 39, 40]. Previous studies from Egypt showed a significant association between HCV seropositivity and the history of schistosomiasis [35, 39]. Baldo et al. and Bafa and Egata demonstrated that the HCV seroprevalence among pregnant women in Italy and Ethiopia is associated with the history of previous abortion [32, 33]. Ephraim et al. demonstrated that the HCV seroprevalence among pregnant women in Ghana is associated with tattooing and needle sharing [30]. This discrepancy between results in different studies might be due to some differences in risk behavior patterns and the predominant routes of transmission in different regions.

Furthermore, we found a significant association between HCV seropositivity and place of residency, so the residents of Khormuj city had significantly higher HCV seroprevalence compared to the residents of other cities. However, this finding cannot be generalized to the pregnant population of Khormuj city due to the low number of the assessed pregnant women. This higher prevalence might be due to the higher prevalence of HCV in the general population of this city. Therefore, residents of Khormuj city can be considered as the main at-risk population in the northern shores of the Persian Gulf. However, more studies among different groups in Khormuj city are required to confirm this issue.

Although HCV infection has been observed throughout the year, the prevalence of HCV infection was more prevalent in those samples collected in May (1.79%). The reason for this observation is not clear. The demand for blood transfusion, elective surgeries, tattooing and piercing for wedding ceremonies, sexual activity, and illicit drug use, which are supposed to be higher in spring, may explain the higher prevalence of HCV infection in May. Overall, the difference in the monthly rate of HCV infection was statistically insignificant. Research to determine the seasonal or monthly pattern of HCV infection is warranted. Moreover, a significantly higher HCV seroprevalence was reported during 2019 (2.2%) than 2018 (0.6%) among pregnant women (OR: 3.61; 95% CI: 1.29–10.08; *P* = 0.014). The lurking epidemic of HCV in Iran, which is increasing silently due to an increase in injecting drug use in the society [41], might expose pregnant women to a greater risk of acquiring hepatitis C in years.

The HCV prevalence of 1.33% observed in pregnant women is lower than the HCV prevalence of 1.98% reported in the diabetic population of Southern Iran [17]. The possible reasons for the higher prevalence of HCV infection in diabetic patients can be their older age and also more frequent visits to medical centers or the probable link between diabetes and HCV infection. In addition, 91% of diabetic patients with positive HCV antibody tests had HCV viremia [17]. While in the present study, 47.37% of anti-HCV-seropositive samples were positive for HCV RNA. Those anti-HCV-positive samples with a negative HCV RNA test are defined as past HCV infection. In this study, the prevalence of HCV viremia among pregnant women was 0.63%. The prevalence of HCV viremia observed in this study is higher than the HCV viremia prevalence of 0.4% reported in the general population of Iran [16] that are shown in supplementary Table 1.

According to the results of the present study, genotype 3a is the only genotype found among HCV-infected pregnant women. The same result has been reported in the diabetic population of southern Iran [17]. It seems that HCV genotype 3a observed in this study follows the predominant genotypic pattern of HCV in this territory. Although genotype 1a is the most prevalent genotype in Iran, genotype 3a has been increasing in recent years. Genotype 1a is more common in older people and genotype 3a in younger people and intravenous drug users. Therefore, injecting drug use is likely to play a significant role in the majority of new infections [41, 42]. However, large-scale epidemiological studies in different geographical locations are required to determine the changes in the distribution pattern of HCV genotypes and to reveal the current common genotypes in different high-risk groups. Moreover, the dose, duration, and type of antiviral therapy can be influenced by the genotypic pattern of HCV infection [42–44].

This study is the first report on the prevalence and the genotypic pattern of HCV infection among pregnant women in the south of Iran, and consecutive recruitment of participants has increased the generalizability of the results to the pregnant women of this region. However, we were not able to confirm the role of injection drug use as the main route of HCV transmission, since a minority of participants respond to the questions regarding injection drug use by their spouses. As another limitation, we could not determine the possible effects of HCV infection on pregnancy outcomes due to the cross-sectional design of the research, which prevents the follow-up of the infection status in infants born to infected mothers. Moreover, in this study, nested-PCR or the two-step PCR technique was used, which increases the sensitivity of diagnosing chronic HCV infections with low virus titers. So, 4 samples were found to be positive in the first round of PCR and 5 samples were positive in the second round of PCR. Besides, positive samples were reexamined to ensure the accuracy of the results and the absence of technical errors or accidental contamination.

## 5. Conclusion

This study reports the HCV prevalence of 1.33% for anti-HCV antibodies and 0.63% for HCV viremia with genotype 3a among pregnant women residents in the northern shores of the Persian Gulf, south of Iran. Given that vertical transmission is possible in women with detectable viremia, therefore, screening of women before pregnancy is recommended to reduce the risk of HCV infection and its complications during pregnancy. According to the results of the present study, the prevalence of HCV infection among pregnant women in this territory is not insignificant and may remain undiagnosed over time due to the asymptomatic nature of chronic hepatitis C infection. Considering the complications of chronic HCV infection, preventive strategies, appropriate precautions, and training programs are also recommended to reduce the incidence and adverse effects of HCV infection in the pregnant population.

## Figures and Tables

**Table 1:** Prevalence of anti-HCV antibodies according to sociodemographic characteristics and qualitative variables among pregnant women in the south of Iran.

	No. of all participants (%): 1425 (100%)	No. of HCV Ab-negative subjects (%): 1406 (98.67%)	No. of HCV Ab-positive subjects (%): 19 (1.33%)	Adjusted OR (95% CI)	*P* value
Age groups (years)					
<20	108 (7.6%)	107 (99.1%)	1 (0.9%)	1.0	
20–24	283 (19.9%)	283 (100.0%)	0 (0.0%)	2.17 (0.27–17.13)	0.994
25–29	503 (35.3%)	493 (98.0%)	10 (2.0%)	1.48 (0.16–13.4)	0.462
30–34	293 (20.6%)	289 (98.6%)	4 (1.4%)	1.69 (0.17–16.53)	0.727
35–39	192 (13.5%)	189 (98.4%)	3 (1.6%)	2.38 (0.15–38.85)	0.648
>39	46 (3.2%)	45 (97.8%)	1 (2.2%)	2.17 (0.27–17.13)	0.543
Place of residence (city)					
Bushehr	616 (43.2%)	609 (98.9%)	7 (1.1%)	1.0	
Borazjan	440 (30.9%)	435 (98.9%)	5 (1.1%)	1.0 (0.32–3.17)	1.000
Ahram	207 (14.50%)	205 (99.1%)	2 (1.0%)	0.85 (0.17–4.12)	0.839
Jam	122 (8.6%)	120 (98.4%)	2 (1.6%)	1.45 (0.29–7.06)	0.646
Khormuj	40 (2.8%)	37 (92.5%)	3 (7.5%)	7.05 (1.75–28.39)	0.006
Ethnicity					
Fars	1283 (90.0%)	1265 (98.6%)	18 (1.4%)	1.0	
Arab	68 (4.8%)	67 (98.5%)	1 (1.5%)	1.05 (0.14–7.98)	0.963
Afghan	62 (4.4%)	62 (100.0%)	0 (0.0%)	0.00	0.997
Turk	12 (0.8%)	12 (100.0%)	0 (0.0%)	0.00	0.999
Stage of gestation					
First trimester	256 (18.0%)	256 (100.0%)	0 (0.0%)	1.0	
Second trimester	194 (13.6%)	193 (99.5%)	1 (0.5%)	0.00	0.995
Third trimester	975 (68.4%)	957 (98.2%)	18 (1.8%)	0.275 (0.04–2.08)	0.211
Number of pregnancies					
One pregnancy	440 (30.9%)	434 (98.6%)	6 (1.4%)	1.0	
Two pregnancies	453 (31.8)	444 (98.0%)	9 (2.0%)	1.466 (0.51–4.15)	0.471
Three pregnancies	332 (23.3)	330 (99.4%)	2 (0.6%)	0.438 (0.09–2.19)	0.314
Four pregnancies	134 (9.4%)	132 (98.5%)	2 (1.5%)	1.096 (0.22–5.49)	0.911
More than four pregnancies	66 (4.6%)	66 (100.0%)	0 (0.0%)	0.00	0.997
History of abortion					
No	934 (65.5%)	921 (98.6%)	13 (1.4%)	1.0	
Yes	231 (16.2%)	227 (98.3%)	4 (1.7%)	1.248 (0.40–3.86)	0.700
Unknown	260 (18.2%)	258 (99.2%)	2 (0.8%)	0.549 (0.12–2.45)	0.432
Education					
Upper diploma	366 (25.7%)	360 (98.4%)	6 (1.6%)	1.0	
Diploma	677 (47.5%)	666 (98.4%)	11 (1.6%)	0.99 (0.36–2.70)	0.986
Under diploma	340 (23.9%)	338 (99.4%)	2 (0.6%)	0.35 (0.07–1.77)	0.207
Uneducated	42 (2.9%)	42 (100.0%)	0 (0.0%)	0.00	0.998
Year					
2018	797 (55.9%)	792 (99.4%)	5 (0.6%)	1.0	
2019	628 (44.1%)	614 (97.8%)	14 (2.2%)	3.61 (1.29–10.08)	0.014
Month					
Oct	113 (7.9%)	112 (99.1%)	1 (0.9%)	1.0	
Nov	102 (7.2%)	102 (100.0%)	0 (0.0%)	0.00	0.997
Dec	112 (7.9%)	111 (99.1%)	1 (0.9%)	1.01 (0.06–16.33)	0.995
Jan	108 (7.6%)	105 (97.2%)	3 (2.8%)	3.20 (0.33–31.25)	0.317
Feb	291 (20.4%)	290 (99.7%)	1 (0.3%)	0.39 (0.02–6.23)	0.502
Mar	248 (17.4%)	246 (99.2%)	2 (0.8%)	0.91 (0.08–10.15)	0.939
Apr	131 (9.2%)	128 (97.7%)	3 (2.3%)	2.63 (0.27–25.59)	0.406
May	168 (11.8%)	162 (96.4%)	6 (3.6%)	4.15 (0.49–34.93)	0.191
June	123 (8.6%)	122 (99.2%)	1 (0.8%)	0.92 (0.06–14.85)	0.952
July	29 (2.0%)	28 (96.6%)	1 (3.4%)	4.00 (0.24–65.95)	0.332
Smoking					
No	836 (58.7%)	821 (98.2%)	15 (1.8%)	1.0	
Yes	62 (4.4%)	61 (98.4%)	1 (1.6%)	0.90 (0.12–6.91)	0.917
Unknown	527 (37.0%)	524 (99.4%)	3 (0.6%)	0.31 (0.09–1.09)	0.068
History of blood injection					
No	892 (62.6%)	877 (98.3%)	15 (1.7%)	1.0	
Yes	18 (1.3%)	17 (94.4%)	1 (5.6%)	3.44 (0.43–27.54)	0.245
Unknown	515 (36.1%)	512 (99.4%)	3 (0.6%)	0.34 (0.10–1.19)	0.092
History of operation					
No	692 (48.6%)	683 (98.7%)	9 (1.3%)	1.0	
Yes	218 (15.3%)	211 (96.8%)	7 (3.2%)	2.52 (0.93–6.84)	0.070
Unknown	515 (36.1%)	512 (99.4%)	3 (0.6%)	0.45 (0.12–1.65)	0.226
History of tattoo					
No	786 (55.2%)	771 (98.1%)	15 (1.9%)	1.0	
Yes	124 (8.7%)	123 (99.2%)	1 (0.8%)	0.42 (0.05–3.19)	0.400
Unknown	515 (36.1%)	512 (99.4%)	3 (0.6%)	0.30 (0.09–1.05)	0.059
History of dentistry					
No	504 (35.4%)	497 (98.6%)	7 (1.4%)	1.0	
Yes	406 (28.5%)	397 (97.8%)	9 (2.2%)	1.61 (0.59–4.36)	0.349
Unknown	515 (36.1%)	512 (99.4%)	3 (0.6%)	0.42 (0.11–1.62)	0.206
History of HBV vaccination					
No	314 (22.0%)	305 (97.1%)	9 (2.9%)	1.0	
Yes	314 (22.0%)	309 (98.4%)	5 (1.6%)	0.55 (0.18–1.65)	0.286
Unknown	797 (55.9%)	792 (99.4%)	5 (0.6%)	0.21 (0.07–0.64)	0.006

**Table 2:** Prevalence of HCV viremia according to sociodemographic characteristics and qualitative variables among pregnant women in the south of Iran.

	No. of all participants (%): 1425 (100%)	No. of HCV RNA-negative subjects (%): 1416 (98.67%)	No. of HCV RNA-positivesubjects (%):9 (0.63%)	Adjusted OR (95% CI)	*P* value
Age groups (years)					
<20	108 (7.6%)	108 (100.0%)	0 (0.0%)	1.0	
20–24	283 (19.9%)	283 (100.0%)	0 (0.0%)	0.00	1.000
25–29	503 (35.3%)	499 (99.2%)	4 (0.8%)	0.00	0.995
30–34	293 (20.6%)	290 (98.98%)	3 (1.02%)	0.77 (0.17–3.49)	0.740
35–39	192 (13.5%)	190 (98.95%)	2 (1.05%)	0.98 (0.16–5.94)	0.985
>39	46 (3.2%)	46 (100.0%)	0 (0.0%)	0.00	0.998
Place of residence (city)					
Bushehr	616 (43.2%)	613 (99.01%)	3 (0.49%)	1.0	
Borazjan	440 (30.9%)	437 (98.9%)	3 (0.68%)	1.4 (0.28–6.98)	0.679
Ahram	207 (14.50%)	206 (99.1%)	1 (0.48%)	0.99 (0.1–9.58)	0.994
Jam	122 (8.6%)	122 (98.4%)	0 (0.0%)	0.00	0.997
Khormuj	40 (2.8%)	38 (92.5%)	2 (5%)	10.75 (1.74–66.3)	0.010
Ethnicity					
Fars	1283 (90.0%)	1275 (99.38%)	8 (0.62%)	1.0	
Arab	68 (4.8%)	67 (98.53%)	1 (1.47%)	2.38 (0.29–19.29)	0.417
Afghan	62 (4.4%)	62 (100.0%)	0 (0.0%)	0.00	0.997
Turk	12 (0.8%)	12 (100.0%)	0 (0.0%)	0.00	0.999
Stage of gestation					
First trimester	256 (18.0%)	256 (100.0%)	0 (0.0%)	1.0	
Second trimester	194 (13.6%)	193 (99.49%)	1 (0.51%)	0.00	0.995
Third trimester	975 (68.4%)	967 (99.18%)	8 (0.82%)	0.00	0.995
Number of pregnancies					
One pregnancy	440 (30.9%)	438 (99.55%)	2 (0.45%)	1.0	
Two and three pregnancies	785 (55.09%)	780 (99.12%)	5 (0.64%)	1.40 (0.27–7.28)	0.686
More than three pregnancies	200 (14.04%)	198 (100.0%)	2 (1.0%)	2.12 (0.31–15.82)	0.429
History of abortion					
No	934 (65.5%)	930 (99.57%)	4 (0.43%)	1.0	
Yes	231 (16.2%)	228 (98.7%)	3 (1.3%)	3.06 (0.68–13.76)	0.145
Unknown	260 (18.2%)	258 (99.23%)	2 (0.77%)	1.80 (0.33–9.89)	0.498
Education					
Upper diploma	366 (25.7%)	363 (99.18%)	3 (0.82%)	1.0	
Diploma	677 (47.5%)	671 (99.11%)	6 (0.89%)	1.08 (0.27–4.35)	0.912
Under diploma	340 (23.9%)	340 (100.0%)	0 (0.0%)	0.00	0.994
Uneducated	42 (2.9%)	42 (100.0%)	0 (0.0%)	0.00	0.998
Year					
2018	797 (55.9%)	794 (99.62%)	3 (0.38%)	1.0	
2019	628 (44.1%)	622 (99.04%)	6 (0.96%)	2.55 (0.64–10.25)	0.186
Month					
Oct	113 (7.9%)	113 (100.0%)	0 (0.0%)	1.0	
Nov	102 (7.2%)	102 (100.0%)	0 (0.0%)	1.00	1.000
Dec	112 (7.9%)	111 (0.11%)	1 (0.89%)	0.00	0.997
Jan	108 (7.6%)	107 (99.07%)	1 (0.93%)	0.96 (0.06–15.61)	0.979
Feb	291 (20.4%)	290 (99.66%)	1 (0.34%)	2.71 (0.17–43.72)	0.482
Mar	248 (17.4%)	247 (99.6%)	1 (0.4%)	0.85 (0.05–13.69)	0.910
Apr	131 (9.2%)	130 (99.31%)	1 (0.76%)	0.53 (0.03–8.48)	0.651
May	168 (11.8%)	165 (98.21%)	3 (1.79%)	0.42 (0.04–4.11)	0.459
June	123 (8.6%)	122 (99.19%)	1 (0.81%)	2.22 (0.23–21.58)	0.493
July	29 (2.0%)	29 (100.0%)	0 (0.0%)	1.00	0.998
Smoking					
No	836 (58.7%)	829 (99.16%)	7 (0.84%)	1.0	
Yes	62 (4.4%)	62 (100.0%)	0 (0.0%)	0.00	0.997
Unknown	527 (37.0%)	525 (99.62%)	2 (0.38%)	0.45 (0.09–2.18)	0.322
History of blood injection					
No	892 (62.6%)	885 (98.3%)	7 (0.79%)	1.0	
Yes	18 (1.3%)	18 ((100.0%)	0 (0.0%)	0.00	0.999
Unknown	515 (36.1%)	513 (99.62%)	2 (0.38%)	0.49 (0.10–2.38)	0.379
History of operation					
No	692 (48.6%)	688 (99.42%)	4 (0.58%)	1.0	
Yes	218 (15.3%)	215 (98.62%)	3 (1.38%)	2.40 (0.53–10.81)	0.254
Unknown	515 (36.1%)	513 (99.61%)	2 (0.39%)	0.67 (0.12–3.67)	0.645
History of tattoo					
No	786 (55.2%)	779 (99.11%)	7 (0.89%)	1.0	
Yes	124 (8.7%)	124 (100.0%)	0 (0.0%)	0.00	0.996
Unknown	515 (36.1%)	513 (99.61%)	2 (0.39%)	0.43 (0.09–2.10)	0.299
History of dentistry					
No	504 (35.4%)	501 (99.4%)	3 (0.6%)	1.0	
Yes	406 (28.5%)	402 (99.01%)	4 (0.99%)	1.66 (0.37–7.47)	0.508
Unknown	515 (36.1%)	513 (99.61%)	2 (0.39%)	0.65 (0.11–3.91)	0.639
History of HBV vaccination					
No	314 (22.0%)	310 (98.73%)	4 (1.27%)	1.0	
Yes	314 (22.0%)	312 (99.36%)	2 (0.64%)	0.50 (0.09–2.73)	0.421
Unknown	797 (55.9%)	794 (99.62%)	3 (0.38%)	0.30 (0.06–1.32)	0.109

## Data Availability

All relevant data are within the paper.

## Supplementary Materials

Supplementary Materials Prevalence of HCV viremia among anti-HCV positive pregnant women in Iran.
