# Supplementary material for: Prevalence and Predominant Genotype of Hepatitis C Virus Infection and Associated Risk Factors among Pregnant Women in Iran
Source: Biomed Res Int. 2021 Sep 18;2021:9294276. doi: 10.1155/2021/9294276 (PMC8476243; doi:10.1155/2021/9294276)
Supplement: Supplementary Materials — Prevalence of HCV viremia among anti-HCV positive pregnant women in Iran. [file 9294276.f1.docx]

**Supplementary table 1. Prevalence of HCV viremia according to** **socio-demographic characteristics and qualitative variables among anti-HCV positive pregnant women in the South of Iran**

|  | **No. of all anti-HCV positive participants (%): 19(100%)** | **No. of HCV RNA negative subjects (%): 10 (52.63%)** | **No. of HCV RNA positive subjects (%):9 (47.37%)** | **P-Value** |
| --- | --- | --- | --- | --- |
| **Age groups (years)** |  |  |  | **0.449** |
| <20 | 1 | 1 | 0 |  |
| 20-24 | 0 | 0 | 0 |  |
| 25-29 | 10 | 6 | 4 |  |
| 30-34 | 4 | 1 | 3 |  |
| 35-39 | 3 | 1 | 2 |  |
| >39 | 1 | 1 | 0 |  |
| **Place of residence**  **(city)** |  |  |  | **0.621** |
| Bushehr | 7 | 4 | 3 |  |
| Borazjan | 5 | 2 | 3 |  |
| Ahram | 2 | 1 | 1 |  |
| Jam | 2 | 2 | 0 |  |
| Khormuj | 3 | 1 | 2 |  |
| **Ethnicity** |  |  |  | **0.474** |
| Fars | 18 | 10 | 8 |  |
| Arab | 1 | 0 | 1 |  |
| Afghan | 0 | 0 | 0 |  |
| Turk | 0 | 0 | 0 |  |
| **Stage of gestation** |  |  |  |  |
| First trimester | 0 | 0 | 0 |  |
| Second trimester | 1 | 0 | 1 |  |
| Third trimester | 8 | 10 | 18 |  |
| **Number of Pregnancies** |  |  |  | **0.258** |
| One pregnancy | 6 | 4 | 2 |  |
| Two and three pregnancies | 11 | 6 | 5 |  |
| More than three pregnancies | 2 | 0 | 2 |  |
| **History of Abortion** |  |  |  | **0.087** |
| No | 13 | 9 | 4 |  |
| Yes | 4 | 1 | 3 |  |
| Unknown | 2 | 0 | 2 |  |
| **Education** |  |  |  | **0.360** |
| Upper diploma | 6 | 3 | 3 |  |
| Diploma | 11 | 5 | 6 |  |
| Under diploma | 2 | 2 | 0 |  |
| Uneducated | 0 | 0 | 0 |  |
| **Year** |  |  |  | **0.51** |
| 2018 | 5 | 2 | 3 |  |
| 2019 | 14 | 8 | 6 |  |
| **Month** |  |  |  | **0.689** |
| Oct | 1 | 1 | 0 |  |
| Nov | 1 | 0 | 1 |  |
| Dec | 3 | 2 | 1 |  |
| Jan | 1 | 0 | 1 |  |
| Feb | 2 | 1 | 1 |  |
| Mar | 3 | 2 | 1 |  |
| Apr | 6 | 3 | 3 |  |
| May | 1 | 0 | 1 |  |
| June | 1 | 1 | 0 |  |
| July | 1 | 1 | 0 |  |
| **Smoking** |  |  |  | **0.509** |
| No | 15 | 8 | 7 |  |
| Yes | 1 | 1 | 0 |  |
| Unknown | 3 | 1 | 2 |  |
| **History of blood transfusion** |  |  |  | **0.509** |
| No | 15 | 8 | 7 |  |
| Yes | 1 | 1 | 0 |  |
| Unknown | 3 | 1 | 2 |  |
| **History of operation** |  |  |  | **0.765** |
| No | 9 | 5 | 4 |  |
| Yes | 7 | 4 | 3 |  |
| Unknown | 3 | 1 | 2 |  |
| **History of tattoo** |  |  |  | **0.509** |
| No | 15 | 8 | 7 |  |
| Yes | 1 | 1 | 0 |  |
| Unknown | 3 | 1 | 2 |  |
| **History of dentistry** |  |  |  | **0.765** |
| No | 7 | 4 | 3 |  |
| Yes | 9 | 5 | 4 |  |
| Unknown | 3 | 1 | 2 |  |
| **History of HBV vaccination** |  |  |  | **0.795** |
| No | 9 | 5 | 4 |  |
| Yes | 5 | 3 | 2 |  |
| Unknown | 5 | 2 | 3 |  |
